# An Efficient, Rapid, and Recyclable System for CRISPR-Mediated Genome Editing in Candida albicans

**DOI:** 10.1128/mSphereDirect.00149-17

**Published:** 2017-04-26

**Authors:** Namkha Nguyen, Morgan M. F. Quail, Aaron D. Hernday

**Affiliations:** a Department of Molecular and Cell Biology, School of Natural Sciences, University of California Merced, Merced, California, USA; b Quantitative and Systems Biology Graduate Program, School of Natural Sciences, University of California Merced, Merced, California, USA; Carnegie Mellon University; Medical University of Vienna; University of Texas Health Science Center at San Antonio; Johns Hopkins University Medical School

**Keywords:** CRISPR, Candida albicans, Cas9, gene addback, gene knockout, genetics, genome editing, markerless

## Abstract

Candida albicans is the most common fungal pathogen of humans. Historically, molecular genetic analysis of this important pathogen has been hampered by the lack of stable plasmids or meiotic cell division, limited selectable markers, and inefficient methods for generating gene knockouts. The recent development of clustered regularly interspaced short palindromic repeat(s) (CRISPR)-based tools for use with C. albicans has opened the door to more efficient genome editing; however, previously reported systems have specific limitations. We report the development of an optimized CRISPR-based genome editing system for use with C. albicans. Our system is highly efficient, does not require molecular cloning, does not leave permanent markers in the genome, and supports rapid, precise genome editing in C. albicans. We also demonstrate the utility of our system for generating two independent homozygous gene knockouts in a single transformation and present a method for generating homozygous wild-type gene addbacks at the native locus. Furthermore, each step of our protocol is compatible with high-throughput strain engineering approaches, thus opening the door to the generation of a complete C. albicans gene knockout library.

**IMPORTANCE** Candida albicans is the major fungal pathogen of humans and is the subject of intense biomedical and discovery research. Until recently, the pace of research in this field has been hampered by the lack of efficient methods for genome editing. We report the development of a highly efficient and flexible genome editing system for use with C. albicans. This system improves upon previously published C. albicans CRISPR systems and enables rapid, precise genome editing without the use of permanent markers. This new tool kit promises to expedite the pace of research on this important fungal pathogen.

## Abstract (precis)

Candida albicans is the major fungal pathogen of humans and is the subject of intense biomedical and discovery research. Until recently, the pace of research in this field has been hampered by the lack of efficient methods for genome editing. We report the development of a highly efficient and flexible genome editing system for use with C. albicans. This system improves upon previously published C. albicans CRISPR systems and enables rapid, precise genome editing without the use of permanent markers. This new tool kit promises to expedite the pace of research on this important fungal pathogen.

## INTRODUCTION

Candida albicans is an opportunistic fungal pathogen that causes a wide range of diseases in humans, and systemic candidiasis is associated with high morbidity and mortality rates. Molecular genetic analysis of this predominantly diploid organism has been limited by a mating cycle that is ill-suited to classical genetics, by the absence of stable plasmids, and by a high degree of genomic plasticity that can lead to frequent aneuploidy. Over the last decade, researchers have relied heavily on the use of a set of auxotrophic strains and heterologous markers for the generation of homozygous gene knockouts (1). While this system represents a significant improvement over prior methods, it requires two sequential rounds of transformation into a doubly (or triply) auxotrophic base strain and takes at least 2 weeks to generate a homozygous gene knockout. Furthermore, the markers in this system cannot be recycled, so the generation of double mutant strains (homozygous deletions in two separate genes) necessitates the use of recyclable marker systems that further extend the time required to make homozygous mutants.

The discovery of programmable RNA-guided DNA nucleases in bacteria and the subsequent development of clustered regularly interspaced short palindromic repeat(s) (CRISPR) genome editing systems have revolutionized genetic engineering in a rapidly expanding number of species (reviewed in references [Bibr B2][Bibr B3][Bibr B5]). These CRISPR genome editing systems rely upon a synthetic guide RNA (gRNA) to direct a programmable nuclease (typically Cas9) to introduce double-stranded breaks (DSBs) at a specific target locus within the genome of a living cell. Since DSBs in genomic DNA are lethal if not repaired, this creates strong selective pressure for repair of the target locus. DNA repair and associated genomic modifications can be mediated via error-prone nonhomologous end joining (NHEJ) or homology-directed repair (HDR). Repair via HDR relies upon the integration of a DNA repair template or donor DNA (dDNA) at the target locus and can be used to introduce modifications ranging from single-base substitutions to integration of large pieces of heterologous DNA. Recently, two groups have reported the development of CRISPR-mediated genome editing systems for use with C. albicans (6–8). Both systems rely upon Cas9-mediated DSBs to stimulate the integration of linear dDNA fragments via homologous recombination at the target locus, and both systems can be used to delete both alleles of a target gene in a single transformation. The first system, developed by Vyas et al., relies upon Cas9 and gRNA expression from a linear DNA fragment that is integrated at the ENO1 locus and integration of unmarked dDNA via HDR at the target locus (6). The second system, developed by Min et al. and revised by Huang and Mitchell, relies upon transient Cas9 and gRNA expression from independent DNA fragments that are not integrated into the genome and integration of selectable markers via HDR at the target locus (7, 8). Although both systems represent significant advances in the state of the art of C. albicans genetic engineering, they each have significant limitations.

We report here the development of an optimized CRISPR genome editing system for use with C. albicans that incorporates four key design features: (i) high-efficiency markerless homozygous genome editing, (ii) rapid cloning-free gRNA generation, (iii) facile marker recycling and removal of CRISPR components, and (iv) a robust standardized protocol that is amenable to high throughput.

## RESULTS

### Plasmid design and system overview.

**FIG 1:**
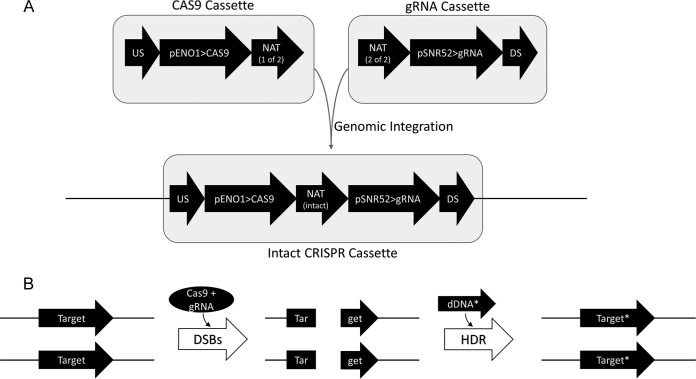
Schematic of CRISPR system components. (A) CRISPR components are transformed into C. albicans as two separate linear DNA fragments that become joined via homologous recombination between the split *NAT* marker elements (*NAT* 1 of 2 and *NAT* 2 of 2). (B) The target locus is modified by the introduction of Cas9-mediated DSBs, followed by integration of a modified target sequence via HDR. The modified target locus is indicated by an asterisk (Target*).

Our optimized C. albicans genome editing system is designed to support markerless homozygous genome editing in virtually any nourseothricin-sensitive C. albicans strain. The CRISPR components—Cas9 and gRNA expression cassettes—are cotransformed along with a linear dDNA fragment in a single transformation. The CRISPR components are designed for integration at a defined locus in the C. albicans genome, and nourseothricin is used to select for stable transformants (Fig. 1A). Coexpression of the Cas9 protein and a target-specific gRNA leads to the introduction of DSBs at the target locus, thus generating a selection for integration of the unmarked dDNA via HDR (Fig. 1B). After confirming the intended target locus modifications, the nourseothricin *N*-acetyltransferase (NAT) marker, along with the Cas9 and gRNA expression cassettes, is removed from the genome. Since no markers are integrated at the target locus and the CRISPR/NAT marker components are recycled after each transformation, this system supports true markerless genome editing.

**FIG 2:**
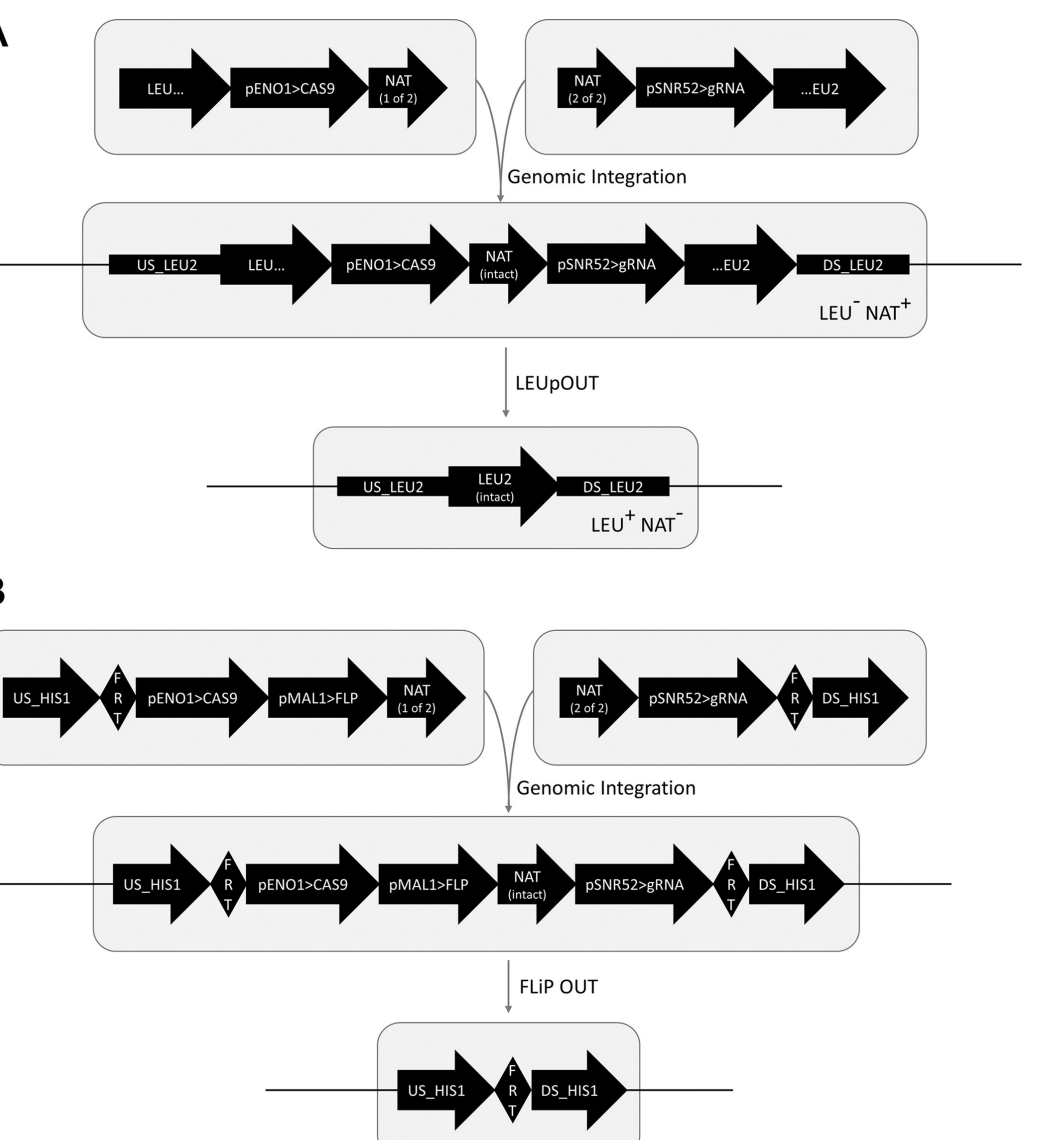
LEUpOUT and HIS/FLP systems for marker/CRISPR recycling. (A) The LEUpOUT-based CRISPR systems are integrated within the *LEU2* ORF and disrupt *LEU2* expression. The resulting direct repeats (indicated by the EU portions of the disrupted *LEU2* ORF) enable spontaneous excision of the CRISPR components via homologous recombination (LEUpOUT). LEU^+^/NAT^−^ transformants can be selected on medium lacking leucine. US_LEU2 and DS_LEU2 represent genomic sequences 5′ and 3′ to the *LEU2* ORF, respectively. (B) The HIS/FLP system is integrated at the *HIS1* locus, replacing one allele of the *HIS1* ORF. CRISPR/NAT excision is mediated by the maltose-inducible FLP recombinase, leaving a single FLP recombinase target (FRT) site in place of the *HIS1* ORF. US_HIS1 and DS_HIS1 represent genomic sequences 5′ and 3′ to the *HIS1* ORF, respectively.

To accommodate CRISPR-mediated editing of all commonly used C. albicans strains, we generated three distinct CRISPR plasmid systems (see Table S1). These systems are (i) the *C.mal* LEUpOUT system, (ii) the *C.alb* LEUpOUT system, and (iii) the HIS-FLP system. The LEUpOUT systems enable rapid and efficient CRISPR/NAT recycling and are recommended for iterative strain engineering projects or high-throughput applications. The *C.mal* and *C.alb* LEUpOUT systems are integrated within the *C. maltosa* and C. albicans *LEU2* open reading frames (ORFs), respectively, and thus generate a NAT^+^/LEU^−^ phenotype when transformed into *LEU2*/Δ*leu2* mutant strains (Fig. 2A). After selecting for nourseothricin-resistant transformants and confirming the intended target locus modifications, CRISPR removal and *LEU2* ORF restoration can be achieved by simple selection on leucine dropout medium (Fig. 2A). We note that all strains that are engineered by the popular Noble and Johnson dual-marker system (1) carry a single copy of the *C. maltosa LEU2* marker and are thus directly compatible with the *C.mal* LEUpOUT system. The *C.alb* LEUpOUT system is compatible with any strain that carries a single copy of the native C. albicans *LEU2* gene. The HIS-FLP system (Fig. 2B) is integrated at the *HIS1* locus and includes a maltose-inducible FLP recombinase system for CRISPR/NAT recycling. Although the CRISPR removal process is more involved with the HIS-FLP system, it is compatible with virtually any nourseothricin-sensitive C. albicans strain, including those that are homozygous for *LEU2*.

10.1128/mSphereDirect.00149-17.6 TABLE S1 Plasmids used for the LEUpOUT and HIS-FLP CRISPR systems. Download TABLE S1, PDF file, 0.01 MB. Copyright © 2017 Nguyen et al. 2017 Nguyen et al. This content is distributed under the terms of the Creative Commons Attribution 4.0 International license.

**FIG 3:**
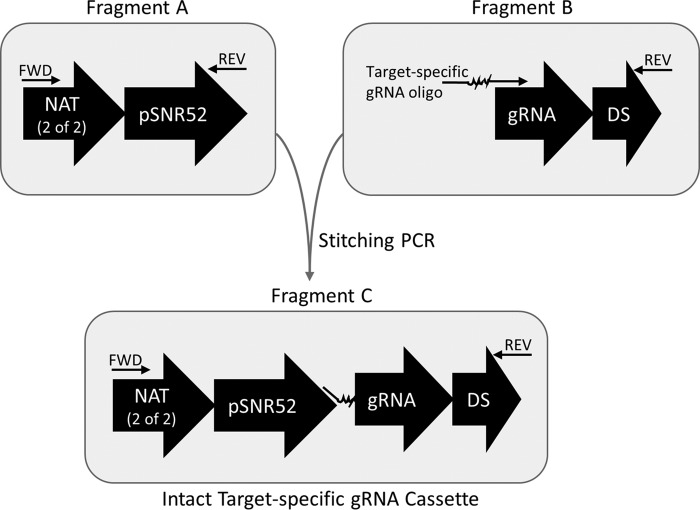
Cloning-free gRNA cassette stitching. A target-specific gRNA oligonucleotide adds the 20-bp targeting sequence (squiggly line) along with homology to the *SNR52* promoter to fragment B. Assembly of fragments A and B creates the intact target-specific gRNA cassette and is mediated by overlap extension stitching PCR. FWD, forward; REV, reverse.

We optimized two methods for the generation of unique target-specific gRNA expression cassettes: cloning-free stitching PCR assembly (Fig. 3) and single-oligonucleotide circular polymerase extension cloning (soCPEC) (see Fig. S1). Both methods rely upon a single 60-mer gRNA oligonucleotide to introduce a unique 20-mer gRNA target sequence within the gRNA expression cassette, and these gRNA oligonucleotides can be used interchangeably with both methods. Since the 5′ and 3′ ends of the gRNA oligonucleotides are invariable and only the internal 20-nucleotide (nt) region is unique to a particular target sequence, we were able to develop standardized gRNA stitching and cloning methods that are unaffected by the various G ⋅ C contents of different target loci. Stitched gRNAs can easily be assembled and transformed into C. albicans within a single day, while our soCPEC method enables rapid, reliable, ligation-free cloning for frequently used gRNAs (see Text S1 in the supplemental material for detailed protocols).

10.1128/mSphereDirect.00149-17.3 FIG S1 Schematic of the soCPEC method of gRNA cloning. A target-specific gRNA oligonucleotide (short linear fragment with an arrow at the 3′ end) is combined with a linear double-stranded entry vector fragment via stitching PCR. Open arrows indicate the first and second steps of CPEC assembly, which occur in subsequent cycles of PCR. The intact cloned gRNA plasmid is recovered after transformation into E. coli and selection on carbenicillin. The sequence represented by gray lines represents a unique 20-bp gRNA target sequence. Download FIG S1, PDF file, 0.2 MB. Copyright © 2017 Nguyen et al. 2017 Nguyen et al. This content is distributed under the terms of the Creative Commons Attribution 4.0 International license.

10.1128/mSphereDirect.00149-17.1 TEXT S1 Detailed supplemental protocols. Download TEXT S1, PDF file, 1 MB. Copyright © 2017 Nguyen et al. 2017 Nguyen et al. This content is distributed under the terms of the Creative Commons Attribution 4.0 International license.

To introduce gene deletions or small-scale modifications at the CRISPR target locus, we recommend the use of synthetic dDNA fragments consisting of annealed complementary oligonucleotides. Since 100 nt is the upper limit for inexpensive oligonucleotides, we limited our synthetic dDNA fragments to this length. In general, dDNA fragments for gene deletions should consist of up to 50 bp of homology to the upstream flank of the target ORF and up to 50 bp of homology to the downstream flank; integration of this dDNA at the target locus results in removal of the target ORF and joining of the upstream and downstream flanks. For integration of larger dDNA fragments, such as gene addbacks, we recommend the use of PCR fragments that contain ~500 bp of upstream and downstream flanking homology to the target locus; shorter homology regions are likely to be functional, but this parameter has not been tested.

### High-efficiency homozygous gene knockouts using cloned gRNA cassettes.

**FIG 4:**
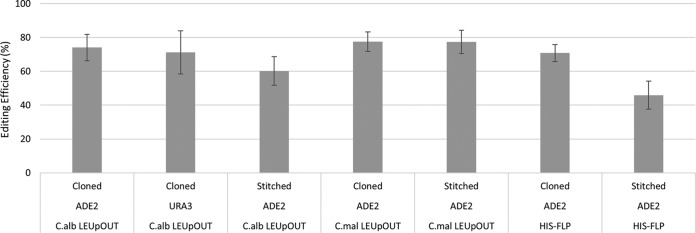
High-efficiency homozygous gene knockouts. All three CRISPR systems generate homozygous gene knockouts with high efficiency with either cloned or stitched gRNA expression cassettes. Variables: cloned versus stitched gRNA expression cassettes, *ADE2* versus *URA3* target loci, *C*.*alb* LEUpOUT versus *C*.*mal* LEUpOUT versus HIS-FLP systems. Editing efficiency represents the average of at least three independent transformations, and error bars represent the standard deviation. On average, each transformation yielded at least 50 total NAT^+^ transformants.

We tested the efficiency of our CRISPR systems by targeting the *ADE2* gene for deletion. Since homozygous *ade2* deletion strains give rise to red colonies, this enables a rapid readout of our targeted homozygous gene knockout efficiency. We observed homozygous *ade2* knockout frequencies of at least 70% with each of our three plasmid systems when using cloned gRNA expression cassettes and 90-bp double-stranded linear dDNA fragments (Fig. 4). We also performed colony PCR to verify that the red colony phenotypes were due to the intended *ADE2* deletions, rather than point mutations or indels that could arise via NHEJ; in all red colonies that were tested by colony PCR, the intended *ade2* deletion was confirmed (data not shown). To confirm that this high efficiency of gene deletion was not unique to the *ADE2* locus, we also used the same strategy to generate homozygous *URA3* deletions. Using the native LEUpOUT system, we observed ~70% efficiency when generating uracil auxotrophic strains (Fig. 4), thus confirming that our system is robust across multiple target loci.

### Cloning-free gRNA assembly enables rapid, high-efficiency gene deletions.

To test our cloning-free system, we generated *ADE2* gRNA expression cassettes via stitching PCR as described in our detailed supplemental protocol (see Text S1). These stitched gRNA cassettes contain the identical *ADE2* gRNA target sequence used in our cloned constructs and yield homozygous *ADE2* knockouts with similar efficiencies (Fig. 4). Furthermore, we routinely observe genome editing efficiencies in excess of 70% across many unique target sites when using the stitched gRNA assembly system for ongoing strain engineering projects (data not shown). Our protocol for generating these stitched gRNAs is standardized, requires only a single unique oligonucleotide, and is easily completed in a single day. In contrast, gRNA cloning methods require a minimum of 3 days to prepare new gRNA constructs for transformation into C. albicans.

### Simultaneous deletion of two distinct target genes in a single transformation.

On the basis of the high efficiency of our homozygous single-gene knockouts and previous observations that transient gRNA expression can be sufficient to drive efficient CRISPR-mediated gene knockouts in Saccharomyces cerevisiae and C. albicans (6, 7, 9), we decided to test the ability of our system to target two distinct genomic loci in a single transformation. We generated stitched gRNA cassettes and annealed 100-mer dDNA fragments to delete *WOR1*, *WOR2*, and *CZF1* and transformed each set independently or in pairs. When transformed individually, each gRNA-dDNA set yielded approximately 80% gene knockout efficiency (see Fig. S2A). When we transformed them in pairs, we observed 20% efficiency with simultaneous deletion of both target genes (see Fig. S2B and C). Since only one of the two gRNA cassettes will be integrated into the genome along with the *CAS9* cassette, this indicates that transient expression of the second gRNA cassette is sufficient to direct Cas9 cutting to the second locus, albeit at reduced efficiency. Although the efficiency of these double-double transformations is lower than that of single-target homozygous gene knockouts and thus necessitates additional colony PCR screening, it is still sufficient for low- to medium-throughput applications and reduces the time required to generate double homozygous mutant strains by half.

10.1128/mSphereDirect.00149-17.4 FIG S2 Colony PCR verification of single and double-double gene knockouts. Colony PCR analysis of *WOR1*, *WOR2*, and *CZF1* single-target knockout transformations (A) and *WOR1*-plus-*WOR2* and *WOR2*-plus-*CZF1* double-double knockout transformations (B). Twenty-four independent colonies from each transformation were tested, using primers specific for the deletion indicated by ←[Δ*gene*] The presence of a band at the mobility indicated confirms that at least one allele of the target gene has been deleted. Selected deletion mutants were further tested for the absence of the target ORF via ORF-check PCRs (C). The presence of a band at the mobility indicated by ←[*GENE*] reveals the presence of at least one allele of the target ORF, while the absence of a band is consistent with homozygous deletion of the target ORF as follows: 1 to 5, *WOR1* single target; 6 to 10, *WOR1*-plus-*WOR2* double double; 11, wild-type control; 12 to 16, *WOR2* single target; 17 to 21, *WOR1*-plus-*WOR2* double double; 22 to 27, *WOR2*-plus-*CZF1* double double; 28, wild-type control; 29 to 33, *CZF1* single target; 34 to 39, *WOR2*-plus-*CZF1* double double; 40, wild-type control. We note that a faint PCR product (indicated by an asterisk) can be observed migrating slightly above the *WOR2* ORF PCR fragment in the Δ*wor2/*Δ*wor2* deletion strain PCRs, as well as in the wild-type control PCRs in panel C; these bands represent nonspecific PCR products and do not indicate the presence of the *WOR2* ORF in our deletion strains. Expected band sizes are as follows: *WOR1*, 0.67 kb; *WOR2*, 0.52 kb; *CZF1*, 0.52 kb; Δ*wor1*, 0.41 kb; Δ*wor2*, 0.86 kb; Δ*czf1*, 0.66 kb. The last four bands in the ladder lanes are 1, 0.75, 0.5, and 0.25 kb. Download FIG S2, PDF file, 0.5 MB. Copyright © 2017 Nguyen et al. 2017 Nguyen et al. This content is distributed under the terms of the Creative Commons Attribution 4.0 International license.

### Rapid construction of homozygous gene addback strains.

A common strain engineering application in the C. albicans field is the generation of gene addbacks, where a wild-type copy of a gene is restored in a mutant background. These strains are used to confirm that the intended deletion, rather than a spontaneous genetic modification, is responsible for the mutant phenotype. Gene addbacks are traditionally generated by integrating a single copy of a wild-type gene, along with a selectable marker, at a nonnative locus (1). This approach is subject to copy number and position-specific effects and can fail to fully restore the wild-type phenotype; in such cases, the interpretation of the addback control is nuanced and may be inconclusive. To improve upon this classic method, we developed a CRISPR-mediated gene addback approach that enables homozygous reintegration of a previously deleted ORF at the native locus.

**FIG 5:**
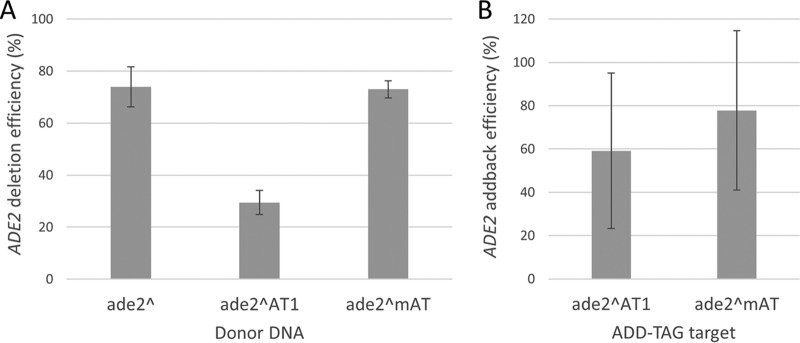
Reintroduction of *ADE2* at the native locus by the ADD-TAG approach. (A) *ADE2* deletion efficiency with standard (ade2^) or ADD-TAG-containing (ade2^AT1, ade2^mAT) dDNAs. Introduction of the 23-bp ADD-TAG1 sequence within the *ADE2* deletion dDNA reduced the gene deletion efficiency by more than 2-fold. The mini-ADD-TAG (mAT) dDNA design restored the *ADE2* deletion efficiency to 73%. Each data point is the average of at least three independent transformations, and the error bars represent the standard deviation. The *ade2* and *ade2^mAT* dDNAs yielded an average of 50 total NAT^+^ transformants per transformation, while the *ade2^AT1* dDNA yielded an average of 20 total transformants per transformation. (B) Efficiency of *ADE2* addback at the native locus with PCR-amplified *ADE2* dDNA and stitched AT1 or mAT gRNA. Efficiency represents the percentage of transformants that were converted to the ADE^+^ (white) colony phenotype. Each data point is the average of at least three independent transformations, and the error bars represent the standard deviation. Addback transformations yielded an average of 15 total NAT^+^ transformants per transformation.

Our gene addback approach relies upon the integration of a unique CRISPR target sequence in place of a deleted ORF, thus creating an ADD-TAG that can be used for subsequent CRISPR-mediated homozygous gene addback transformations. To test the utility of this system, we created two distinct *ADE2* deletion strains; one carrying a 23-bp ADD-TAG1 sequence (AT1 [CGAGACGAGTGCTCGACATGAGG]) in place of the *ADE2* ORF and the second carrying a mini-ADD-TAG sequence (mAT [GG]). The first approach integrates a completely novel CRISPR target site (20-bp gRNA target plus AGG PAM motif), while the second approach relies upon the junction between the upstream flank of the deleted *ADE2* ORF and the introduced GG dinucleotide to create a unique CRISPR target site; CATATACAAGCACTACACATAATG(ADE2ORF)-3′ flank is converted to CATATACAAGCACTACACATAGG-3′ flank. Since both approaches utilized annealed 100-mer dDNA fragments to make the initial *ade2* deletion and integrate the ADD-TAG sequence, the first approach was limited to a total of 77 bp of homology to the *ADE2* locus (split between upstream and downstream flanking homology), while the second approach enabled the use of longer flanks, for a total of 98 bp of homology to the *ADE2* locus. As indicated in Fig. 5A, the shorter homology arms of the AT1 design appear to compromise deletion efficiency, while the longer homology arms of the mini-ADD-TAG design supported highly efficient *ade2* deletion transformations.

To reintroduce *ADE2* at the native locus, we transformed our two *ade2*^ADD-TAG strains with gRNAs specific to each ADD-TAG sequence, along with dDNA composed of a 2.8-kb PCR fragment that encompasses the native *ADE2* ORF plus ~500 bp each of upstream and downstream flanking homology. Using 100 µl of PCR-amplified *ADE2* dDNA, we observed >50% efficiency in the conversion of homozygous *ade2* deletion mutants (red) to the wild-type (white) phenotype (Fig. 5B). Colony PCR analysis of representative white colonies was consistent with homozygous restoration of the *ADE2* locus (see Fig. S3), thus confirming that our mini-ADD-TAG approach can support both high-efficiency homozygous deletion and subsequent addback of a target gene at the native locus. We also note that the use of unique ADD-TAG sequences for each distinct gene deletion strain would generate DNA signature-tagged strains and thus enable the analysis of pooled mutants.

10.1128/mSphereDirect.00149-17.5 FIG S3 Colony PCR verification of the ADD-TAG approach. Colony PCR confirmation of *ade2* homozygous deletion and *ADE2* homozygous addback by the ADD-TAG approach. Colony numbers at the top of each gel represent the following: 1 to 16, independent white colonies isolated from transformation of an *ade2*^mAT/*ade2*^mAT strain (AHY980) with *ade2*^mAT-specific gRNA and *ADE2* plus flanks dDNA; 17 and 18, independent colonies of *ade2*^mAT/*ade2*^mAT strain AHY980. Expected colony PCR bands are indicated as follows: *ade2*^mAT yields a 0.57-kb fragment if the *ADE2* ORF has been replaced with the mini-ADD-TAG sequence, US-*ADE2* yields a 0.71-kb fragment if the wild-type *ADE2* ORF is present at the *ADE2* locus (checking the 5′ side of the ORF), and DS-*ADE2* yields a 0.77-kb fragment if the wild-type *ADE2* ORF is present at the *ADE2* locus (checking the 3′ side of the ORF). The last four bands in the ladder lanes are 1, 0.75, 0.5, and 0.25 kb. Colonies 17 and 18 are confirmed positive for the mAT integration in place of the *ADE2* ORF and are consistent with homozygous deletion of *ADE2*. All 16 of the white colonies tested (colonies 1 to 16) are consistent with removal of the mAT sequence and homozygous addback of the *ADE2* ORF at the *ADE2* locus. Download FIG S3, PDF file, 0.4 MB. Copyright © 2017 Nguyen et al. 2017 Nguyen et al. This content is distributed under the terms of the Creative Commons Attribution 4.0 International license.

## DISCUSSION

We present the development of an optimized system for CRISPR-mediated genetic engineering in C. albicans. Our system expands upon the strengths of the previously developed C. albicans CRISPR systems while avoiding the limitations of each. While all three systems enable users to generate homozygous gene knockouts in C. albicans in a single transformation, there are significant differences in the design and implementation of these systems. While we believe that our system represents the optimal combination of efficiency and flexibility, we present an evaluation of the strengths and weaknesses of each system as a resource for prospective users to evaluate which system best suits their needs. This comparison is discussed below and summarized in Table 1.

**TABLE 1:** Comparison of C. albicans CRISPR systems^a^

Attribute	Nguyen et al.	Min et al./Huang and Mitchell	Vyas et al.
Cloning-free gRNA assembly	+	+	−
Markerless genome editing at target locus	+	−	+
Recyclable markers	+	±^b^	+
Edited strains do not retain CRISPR components	+	+	−
Target-independent gRNA stitching protocol	+	−	NA^c^

a Comparison of key attributes of the three CRISPR systems that have been developed for use with C. albicans. Advantages (+) and limitations (−) are indicated.

b Marker excision in the Huang and Mitchell system is limited to subsequent transformations and requires the integration of a distinct marker at a second target locus.

c NA not applicable.

The Vyas et al. system enables markerless precision editing of the target locus and relies upon Cas9 and gRNA expression from a large linear DNA fragment that becomes integrated into the *ENO1* locus during CRISPR transformation. Modification of the target locus is stimulated by Cas9-mediated DSBs, which provide selective pressure for the integration of unmarked dDNA fragments via HDR. A key limitation of the Vyas et al. system is that targeting of a new genetic locus requires ligation-mediated cloning of the 20-bp gRNA targeting sequence into large (~14-kb) unstable plasmids. Furthermore, although the marker and gRNA cassettes can be excised by conditional expression of the FLP recombinase, the Vyas et al. system generates a permanent disruption of one allele of *ENO1* by leaving the *CAS9* expression construct behind. Since *ENO1* encodes the major protective antigen of C. albicans, *in vivo* analysis of mutants that are generated with this system will require careful attention to the use of isogenic strains; this could be particularly important following two or more rounds of CRISPR transformation, since some strains may carry homozygous *ENO1* disruptions. Subsequent unpublished revisions of this system have avoided some of these limitations; however, the revised system is still limited by a lengthy gRNA cloning process and inefficient marker recycling.

The system developed by Min et al. bypasses the requirement for cloning of new gRNA sequences in Escherichia coli and avoids the potential issues associated with integration into the *ENO1* locus. Instead, this system relies upon transient Cas9 and gRNA expression from linear DNA fragments that do not become integrated into the C. albicans genome. Rather than selecting for a marked gRNA/Cas9 expression cassette, Min et al. apply selection for marked dDNA cassettes that are designed for integration at the target locus after it is cut by Cas9. Although the incorporation of a cloning-free method for generating new gRNAs greatly simplifies the process of making gene deletions, the reliance on marked dDNAs comes at a cost; users are unable to perform markerless precision genome editing such as SNP swaps, the introduction of point mutations or binding-site mutants, or any other small-scale edits. Furthermore, since the Min et al. system utilizes nonrecyclable markers, it is subject to the same shortage of markers that affects traditional methods of strain engineering in C. albicans. In a follow-up to the Min et al. publication, Huang and Mitchell demonstrated a system that enables marker excision in subsequent transformations (8). Briefly, the marker used to delete target gene 1 can be excised during a subsequent transformation that simultaneously integrates a distinct marker in place of target gene 2. While this system enables rapid serial gene deletion, it is still incompatible with true markerless genome editing, requires the use of two distinct selectable markers, and involves alternation between markers, thus complicating the creation of isogenic control strains.

Our CRISPR system streamlines the processes of gRNA and dDNA assembly while simultaneously supporting markerless genome editing and iterative strain engineering projects. While the previous systems rely upon lengthy gRNA cloning procedures or the integration of markers at the target locus, our system enables researchers to quickly perform homozygous genome editing in any nourseothricin-sensitive C. albicans strain without introducing any permanent markers. Since strains that are constructed with our system are prototrophic and nourseothricin sensitive, they can be used immediately for any downstream analysis or for subsequent rounds of CRISPR transformations. Furthermore, our gRNA assembly protocols have been standardized, thus allowing for robustness across multiple target loci without the need for target-specific optimization of gRNA assembly. To edit a specific genetic locus requires only three unique synthetic oligonucleotides (one gRNA oligonucleotide and two complementary dDNA oligonucleotides) and requires no cloning, and all necessary reagents can be prepared and transformed into C. albicans within a single day. Although we have not yet validated our system with the use of high-throughput methods, such as automated liquid-handling or colony-picking robots, we designed every step of our protocol, including the LEUpOUT process of marker excision, to be easily automated. We also demonstrate a proof-of-principle method for facile engineering of signature-tagged knockout strains and for subsequent restoration of wild-type alleles at the native locus. Together, these features enable rapid, flexible, and iterative strain engineering projects with C. albicans and open the door for high-throughput generation of a complete gene knockout library.

## MATERIALS AND METHODS

### Strains and media.

All of the C. albicans strains used in this study were derived from strain SC5314, and a complete list of the strains is provided in Table S2. Nourseothricin-sensitive C. albicans strains were cultured in yeast extract-peptone-dextrose (YPD) liquid medium at 30°C and harvested at an optical density at 600 nm between 0.5 and 0.8 prior to transformation by a modified version of the standard lithium acetate protocol (10); see our detailed protocol in Text S1. After recovery in liquid YPD for 5 h, nourseothricin-resistant transformants were selected on YPD agar supplemented with 200 µg/ml nourseothricin (GoldBio). Subsequent removal of the CRISPR components was performed by single-colony isolation on synthetic defined (SD) agar medium minus leucine for the LEUpOUT method or by culturing overnight in YP-maltose liquid medium, followed by screening on YPD agar supplemented with 25 µg/ml nourseothricin for the FLP recombinase-mediated method (see Text S1 for details). The generation of homozygous *URA3* deletion strains was confirmed by patching to SD minus uracil versus YPD plates; both medium types were supplemented with 200 µg/ml nourseothricin to maintain selection for strains that had integrated the CRISPR components. All E. coli strains were derived from DH5α and cultured at 37°C in LB medium supplemented with 100 µg/ml carbenicillin.

10.1128/mSphereDirect.00149-17.7 TABLE S2 Strains used in this study. Download TABLE S2, XLSX file, 0.02 MB. Copyright © 2017 Nguyen et al. 2017 Nguyen et al. This content is distributed under the terms of the Creative Commons Attribution 4.0 International license.

### Plasmids and DNA.

A list of all of the plasmids generated for this study is included in Table S3, and all of these plasmids and associated sequence files are publicly available through Addgene. For a complete list of the oligonucleotides used in this study, see Table S4. The CaCas9 and gRNA expression cassettes were obtained via PCR amplification from pV1081 (6), the maltose-induced FLP recombinase cassette was isolated from pSFS2a (11), and the *AgNAT* cassette was isolated from pCJN542 (12). All C. albicans genomic DNA fragments were PCR amplified from SC5314 genomic DNA, and the *C. maltosa LEU2* fragments were amplified from pSN40 (1). Phusion polymerase (Thermo Scientific) was used to amplify all fragments that were used for cloning or for direct transformation into C. albicans. DreamTaq Green DNA polymerase (Thermo Scientific) was used for colony PCR applications. Plasmids were assembled by *in vivo* gap repair in E. coli (13) or by circular polymerase extension cloning (14). Our PCR stitching and soCPEC methods for gRNA assembly are described in Text S1. Cas9 expression plasmids and cloned gRNA expression plasmids were digested with FastDigest MssI (Thermo Scientific) to release linear integration fragments prior to transformation into C. albicans, and stitched gRNA fragments were assembled and amplified with Phusion polymerase. Synthetic dDNA fragments were designed as complementary oligonucleotides and annealed in 1× FastDigest buffer with a 0.1°C/s ramp from 99°C to 65°C in a thermal cycler with a heated lid. The 90-bp *ADE2* deletion dDNA consisted of two 45-bp regions of homology to the upstream and downstream flanks, respectively, of the *ADE2* ORF. The *WOR1*, *WOR2*, and *CZF1* ORF deletion dDNAs were 100 bp in length, with 50 bp each of upstream and downstream flanking homology. The ADD-TAG1 dDNA design for *ADE2* contained 39 and 38 bp of upstream and downstream homology, respectively, flanking the 23-bp ADD-TAG sequence. The mini-ADD-TAG dDNA for *ADE2* contained 52 and 46 bp of upstream and downstream homology, respectively, flanking the 2-bp GG insertion that introduced a PAM motif and thus generated a new CRISPR target site. The *ADE2* addback dDNA was generated by PCR amplification of the native *ADE2* locus with oligonucleotides AHO1179 and AHO1182 as primers. The resulting PCR product contains 519 bp of upstream homology and 542 bp of downstream homology, respectively, to the *ADE2* locus. CRISPR target sequences were identified with the Design and Analyze Guides tool of Benchling (San Francisco, CA) with the following settings: design type, single guide; guide length, 20 bp; genome, CA22 (C. albicans SC5314 [diploid]); PAM, NGG. Custom gRNA oligonucleotides for stitching or soCPEC cloning were designed by adding the following flanking sequences: 5′-CGTAAACTATTTTTAATTTG(20-bp target sequence)GTTTTAGAGCTAGAAATAGC-3′. We also provide an excel calculator (see Text S2) that can be used to automatically convert 20-bp gRNA target sequences into custom gRNA oligonucleotide sequences that are compatible with our CRISPR protocols.

10.1128/mSphereDirect.00149-17.8 TABLE S3 Plasmids used in this study. Download TABLE S3, XLSX file, 0.3 MB. Copyright © 2017 Nguyen et al. 2017 Nguyen et al. This content is distributed under the terms of the Creative Commons Attribution 4.0 International license.

10.1128/mSphereDirect.00149-17.9 TABLE S4 Oligonucleotides used in this study. Download TABLE S4, XLSX file, 0.01 MB. Copyright © 2017 Nguyen et al. 2017 Nguyen et al. This content is distributed under the terms of the Creative Commons Attribution 4.0 International license.

10.1128/mSphereDirect.00149-17.2 TEXT S2 gRNA oligonucleotide calculator. Download TEXT S2, XLSX file, 0.01 MB. Copyright © 2017 Nguyen et al. 2017 Nguyen et al. This content is distributed under the terms of the Creative Commons Attribution 4.0 International license.
