# Supplementary material for: An Efficient, Rapid, and Recyclable System for CRISPR-Mediated Genome Editing in Candida albicans
Source: mSphere. 2017 Apr 26;2(2):e00149-17. doi: 10.1128/mSphereDirect.00149-17 (PMC5422035; doi:10.1128/mSphereDirect.00149-17)
Supplement: TABLE S1 [file sph002172275st3.pdf]

Table S1

CAS9/gRNA integration site	Marker/CRISPR removal strategy	*CAS9* plasmid	gRNA plasmid (intact)	gRNA plasmid* (1of2)	gRNA plasmid* (2of2)
C.alb LEU2	LEUpOUT	pADH137	pADH118-#	pADH110	pADH119
C.mal LEU2	LEUpOUT	pADH140	pADH143-#	pADH110	pADH139
C.alb HIS1	FLP	pADH99	pADH100-#	pADH110	pADH147

*plasmids used for cloning-free gRNA stitching protocol
