# Supplementary figures and images for: An Efficient, Rapid, and Recyclable System for CRISPR-Mediated Genome Editing in Candida albicans
Source: mSphere. 2017 Apr 26;2(2):e00149-17. doi: 10.1128/mSphereDirect.00149-17 (PMC5422035; doi:10.1128/mSphereDirect.00149-17)

Figure S1

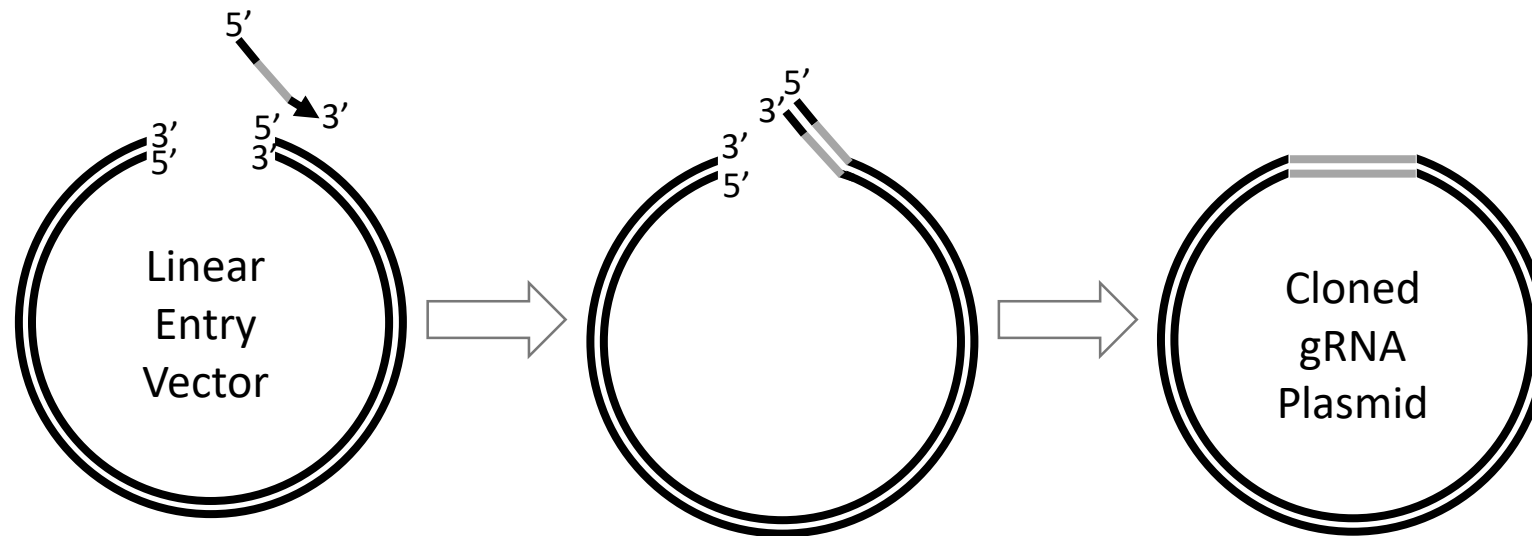

Supplement: FIG S1 [file sph002172275sf7.pdf]

Figure S3

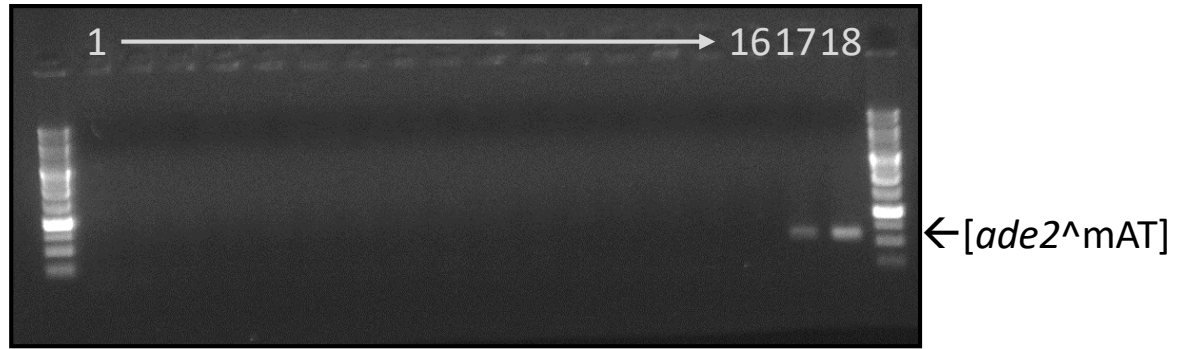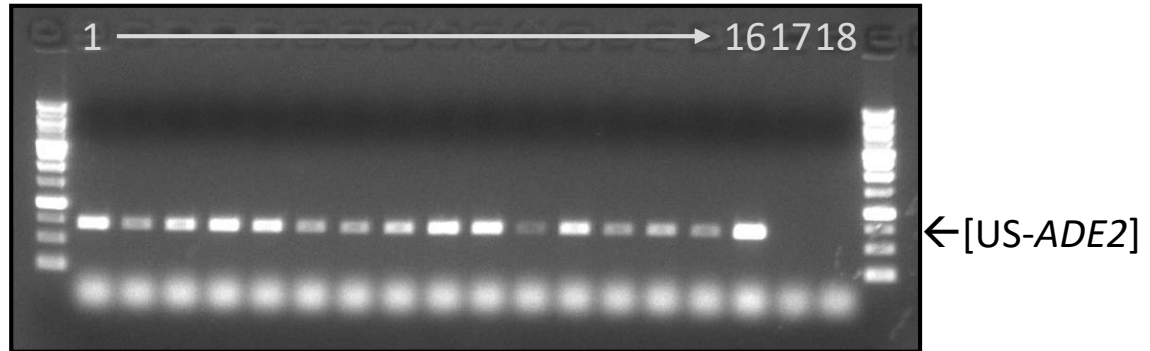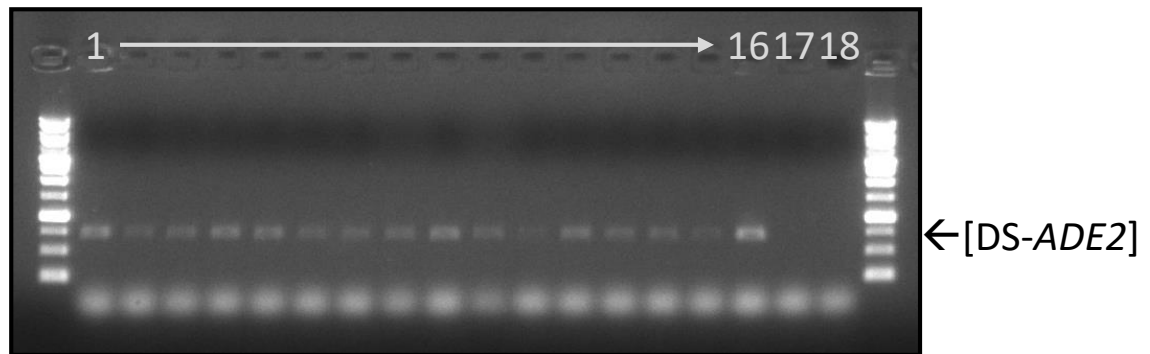

Supplement: FIG S3 [file sph002172275sf9.pdf]
