# Supplementary material for: An Efficient, Rapid, and Recyclable System for CRISPR-Mediated Genome Editing in Candida albicans
Source: mSphere. 2017 Apr 26;2(2):e00149-17. doi: 10.1128/mSphereDirect.00149-17 (PMC5422035; doi:10.1128/mSphereDirect.00149-17)
Supplement: TEXT S1 [file sph002172275s1.pdf]

Herndey Lab *C. albicans* CRISPR System

Background:

This document covers the plasmids and protocols used for the Hernday lab *Candida albicans* CRISPR system. This system allows the user to edit virtually any target locus in the *C. albicans* genome, without the need to integrate a marker gene at the target locus. Thus, even a single nucleotide change could be introduced at the target locus. The system consists of three key components: 1) CAS9, which encodes an RNA-directed nuclease 2) A guide-RNA (gRNA) that directs Cas9 to create double-stranded breaks at a specific target locus in the genome, and 3) Donor DNA (dDNA) that, via homologous recombination, will repair the double-stranded breaks introduced by Cas9+gRNA. In order to modify both alleles of the target locus, it is imperative that the gRNA target sequence and the neighboring NGG "PAM motif" are present in both alleles. It is also possible to generate homozygous modifications at two distinct target loci in a single transformation if two different gRNA sequences are expressed simultaneously; although the efficiency is lower than that of a single-locus modification, it is possible to isolate double homozygous mutants. All of the Hernday Lab CRISPR plasmids utilize the nourseothricin-resistance marker (NAT) to select for stable expression of Cas9 and the gRNA, so all strains must be NAT-sensitive before transformation. After selecting for NAT-resistant transformants and confirming the intended genetic modification(s) at the target locus (or loci), then the CAS9, NAT, and gRNA expression cassettes should be removed from the genome. Most of the Hernday Lab CRISPR plasmids are designed around a "LEUpOUT" technology that enables the user to easily remove all of the CRISPR components by simply re-streaking the strain onto media lacking leucine. This "LEUpOUT" system is much faster and easier than the traditional SAT1/FLP approach, but it does require that your starting strain is heterozygous for the LEU2 ORF. Two distinct sets of LEUpOUT plasmids have been designed: the "*C.alb*" set supports the use of strains that carry a single copy of the native *C. albicans* LEU2 ORF, while the "*C.mal*" system supports the use of strains that carry a single copy of the *C. maltosa* LEU2 marker that is used in the Noble and Johnson gene knockout system (1). To support the use of strains that are not LEU2 heterozygotes, we also include a "HIS-FLP" system that integrates at the *C. albicans* HIS1 locus and enables CAS9, NAT, and gRNA removal via use of an inducible FLP recombinase. Table 1 outlines which plasmids are designed for use with each approach. The plasmids indicated with an asterisk are used with a cloning-free method to generate gRNA expression cassettes, as described below. The "-#" suffix on the intact gRNA plasmid numbers is used to indicate a particular gRNA target sequence; for example, pADH118-2 and pADH143-2 are cloned gRNA expression cassettes, both of which carry the same 20bp targeting sequence for the ADE2 locus. We recommend using this numeric system, along with our gRNA oligo calculator spreadsheet (supplemental file S5), to keep track of unique gRNAs and their target loci.

Table 1: CRISPR plasmid selection guide

CAS9/gRNA integration site	Marker/CRISPR removal strategy	CAS9 plasmid	gRNA plasmid (intact)	gRNA plasmid* (1of2)	gRNA plasmid* (2of2)
C.alb LEU2	LEUpOUT	pADH137	pADH118-#	pADH110	pADH119
C.mal LEU2	LEUpOUT	pADH140	pADH143-#	pADH110	pADH139
C.alb HIS1	FLP	pADH99	pADH100-#	pADH110	pADH147

Design:

This system takes advantage of a split-marker approach, where the CAS9 and gRNA expression constructs, each of which contains overlapping portions of the NAT marker, are carried on two separate plasmids. During the transformation process, recombination between the NAT 1of2 and NAT 2of2 fragments reconstitutes a functional NAT marker, thus enriching for transformants that undergo efficient homologous recombination. Figure 1 represents the general schematic of the LEUpOUT-enabled CRISPR system, including an "intact" gRNA expression plasmid (scissors indicate MssI cut sites). If using a pre-cloned "intact" gRNA plasmid, then simply digest both the gRNA plasmid and the accompanying CAS9 plasmid with MssI to release the linear transformation fragments, and transform into *C. albicans* along with your donor DNA fragment(s). The gRNA and CAS9 fragments, along with the reconstituted NAT marker, will integrate within the LEU2 ORF, causing disruption of the single copy of LEU2 in a LEU2/Δleu2 heterozygote (see figure 2). The LEU2 1of2 and LEU2 2of2 fragments contain direct repeats of the central portion of the LEU2 ORF, allowing for spontaneous homologous recombination between LEU2 1of2 and LEU2 2of2. Since integration of the CRISPR components at LEU2 is unstable in the absence of NAT selection, all of the CRISPR components, including the NAT marker, can

Figure 1: Overview of LEUpOUT-enabled plasmid designs

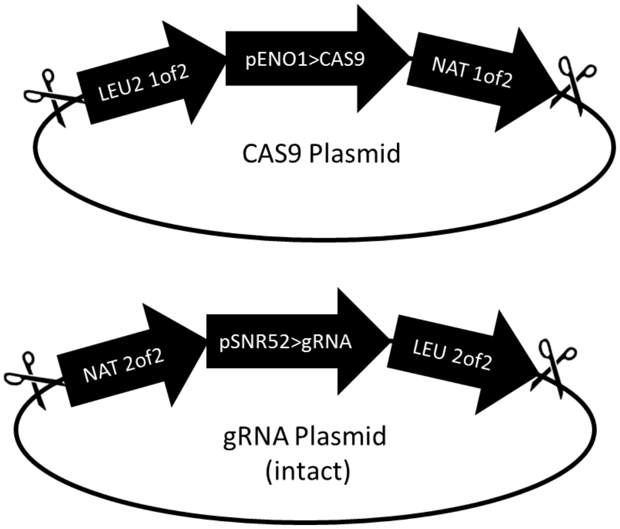

Figure 2: Integration and LEUpOUT of CRISPR components at the LEU2 locus

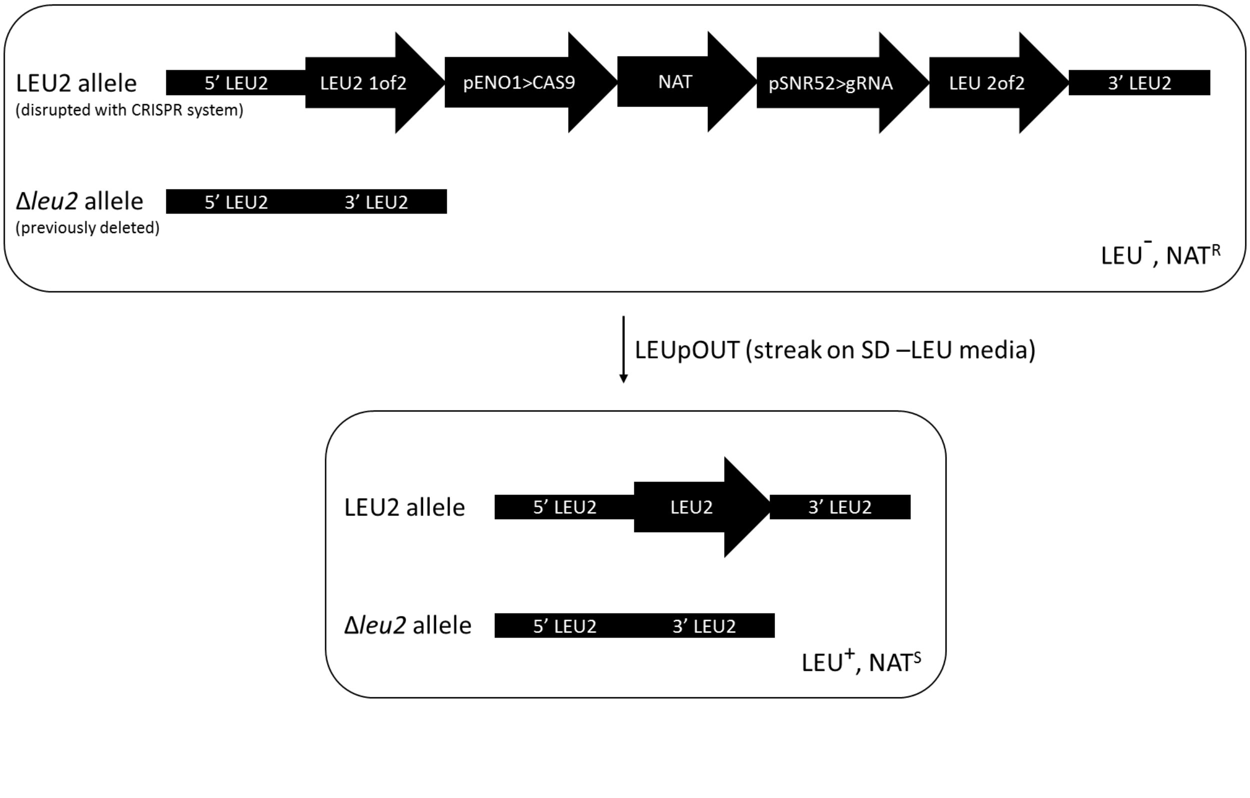

be removed from the LEU2 locus by simply streaking onto minimal media lacking leucine. At this point the strain is now LEU+ and NAT^S, and can be transformed again with the same system using different gRNA sequence to make additional genomic modifications. Note: if transforming with two distinct gRNA expression cassettes, each of which targets a unique genomic locus, only one of the two gRNA expression cassettes will become integrated at LEU2, while the other gRNA will be expressed transiently before being degraded.

A cloning-free method is available to generate custom gRNA cassettes. This approach relies on stitching PCR to fuse the SNR52 promoter from gRNA plasmid 1of2 to the structural gRNA coding sequence carried on gRNA plasmid 2of2 (see figure 3). The two fragments are bridged by a custom gRNA oligo which contains a unique 20bp sequence for the desired genomic target locus. Note that gRNA plasmid 1of2 (pADH110) is common to all of our CRISPR systems, and that the AHO1096/1098 PCR fragment "A" can be used as a universal accepter for any gRNA plasmid 2of2 fragment "B" in the full-length fragment "C" PCR stitching reaction. Targeting a new site in the genome requires only a single custom gRNA oligo and two standardized PCR protocols to create a new stitched gRNA expression fragment "C". No cloning, gel-purification, or other DNA cleanup steps are required; simply transform the stitched gRNA fragment "C" directly into *C. albicans* along with the appropriate MssI-digested CAS9 plasmid and your donor DNA.

Figure 3: Cloning-free PCR stitching of custom gRNA expression cassettes

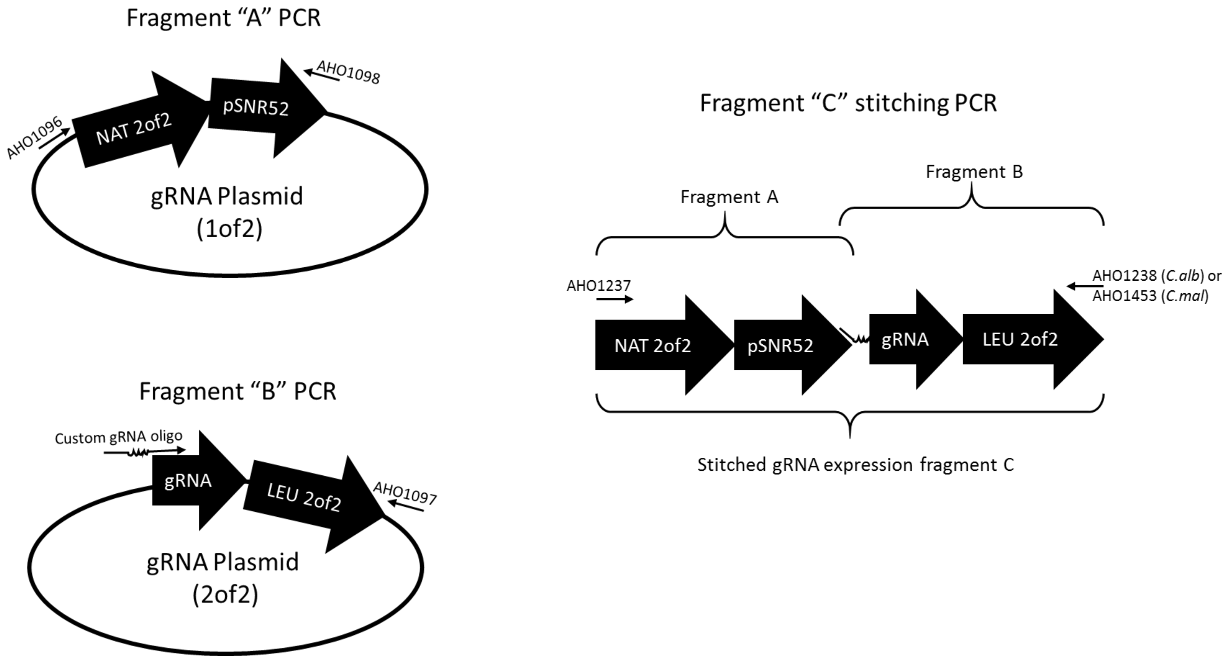

Protocols:

The following protocols are optimized for use with this CRISPR system. Please read CAREFULLY prior to using this system, and note that changes relative to traditional methods are included to improve efficiency. Each step has been optimized with the specific reagents listed in this protocol, and use of any alternate reagents may significantly compromise performance. Note that transformation efficiency is highly dependent on the growth phase of *C. albicans*, and it is critical to use actively growing cultures that are below an OD_{600} of 1. Also, ensure that all reagents to be used are fresh.

Key reagents:

FastDigest MssI (Thermo Scientific catalog # FERFD1344)

Phusion Polymerase (Thermo Scientific catalog # F534)

1M Lithium Acetate, pH7.0 (made using: MP Biomedicals catalog # 0215525683)

50% w/v PEG 3350 (made using: J.T. Baker catalog # JTU221)

dNTP mix, 10 mM each (NEB catalog # N0447)

Salmon sperm DNA (Invitrogen catalog # 15632011)

FastDigest DpnI *for CPEC cloning of gRNAs (Thermo Scientific catalog # FERFD1704)

DreamTaq Green Polymerase *for colony PCR (Thermo Scientific catalog # EP0713)

5M Betaine *for colony PCR (made using: Fisher Scientific catalog # AC20424)

Plasmid digestion with MssI:

Plasmids should be digested with MssI in 1x FastDigest Buffer at a final concentration of 200ng/uL. The sample reactions indicated below are sufficient for a single transformation, and should be scaled accordingly if multiple transformations will be performed. All digests should be incubated at <u>37°C for a minimum of 30 minutes</u> prior to transformation into *C. albicans*. To avoid excessive evaporation, perform digests in a thermocycler with heated lid. The digested plasmids should be transformed directly into *C. albicans* <u>without any cleanup</u>.

- Single-plasmid digest, add:
  1. 2ug of plasmid DNA + H_2O to 8.5uL total volume
  2. 1ul 10X FastDigest buffer
  3. 0.5uL MssI

- Dual-plasmid digest, add:
  1. 2ug of <u>each</u> plasmid + H_2O to 17uL total volume
  2. 2ul 10X FastDigest buffer
  3. 1uL MssI

Guide RNA oligo design:

CRISPR target sequences can be designed using the "Design and Analyze Guides" tool on Benchling (www.benchling.com) with the following settings: Design Type, single guide; Guide Length, 20bp; Genome, CA22 (Candida albicans SC5314 (Diploid)); PAM, NGG. With the "Design CRISPR" tab open, select the target region of your sequence to which you would like to design a gRNA, and click the green "+" icon. Choose your gRNA target sequence based on the optimal combination of "On-Target Score", "Off-Target Score", and location (if relevant). Once you have selected a target sequence, click the check box next to the target of choice, and click to highlight the directional arrow representing this target in the "Sequence Map" pane. Copy the target sequence, paying close attention to directionality (use "copy reverse complement" for "reverse" arrows). After selecting a 20bp target sequence, add the follows flanking sequences:

5'-CGTAAACTATTTTTAATTTG(your target sequence)GTTTTAGAGCTAGAAATAGC-3'

The CRISPR gRNA Calculator excel file can be used to quickly convert 20bp gRNA target sequences into custom gRNA oligo sequences that are compatible with our CRISPR protocols, and provides a convenient format for tracking gRNA oligos and their respective targets.

Cloning of new "intact" gRNA plasmids:

This protocol utilizes circular polymerase extension cloning (CPEC) to introduce custom 20bp gRNA target sequences into intact circular gRNA expression plasmids (pADH118-#, pADH143-#, or pADH100-#). This technique utilizes the same custom gRNA primer that is used for the cloning-free stitching of gRNA expression cassettes (described below).

- Preparation of linear "entry vector":
  - A. PCR Mix (makes enough for several CPEC cloning reactions):
    1. 75.5ul H_2O
    2. 20ul 5x Phusion HF buffer
    3. 2ul dNTP mix (10 mM each dNTP)
    4. 1ul template plasmid (pADH118, 143, or 100) (1ng/ul)
    5. 0.5ul AHO1098 (100uM)
    6. 0.5ul AHO1099 (100uM)
    7. 0.5ul Phusion polymerase
  - B. PCR Cycling Conditions:
    1. 98°C, 30sec
    2. 98°C, 20sec
    3. 58°C, 20sec
    4. 72°C, 30sec
    5. Return to step 2 for a total of 30 cycles
    6. End
  - C. DpnI digestion:
    1. Add 10ul 10x FastDigest buffer
    2. Add 0.5ul FastDigest DpnI
    3. Digest at least 30min at 37°C
  - D. Gel extraction: Follow standard protocols and isolate 4.1kb fragment
- soCPEC cloning of guide RNA plasmid:
  - A. PCR Mix:

1. 4uL 5x Phusion HF Buffer
2. 0.4uL 10mM ea. dNTPs
3. 0.2uL Phusion Polymerase
4. 200ng linear "entry vector"
5. 3ul 100nM custom gRNA oligo (2:1 molar ratio to entry vector)
6. H_2O to 20ul final

B. Cycling Conditions**:**
1. 98°C, 30sec
2. 98°C, 10sec
3. Standard ramp to 68°C
4. Slow ramp from 68°C to 50°C, 0.1^{o}C/sec
5. 50°C, 30sec
6. 72°C, 1min 30sec
7. Return to step 2 for a total of 10 cycles
8. 72°C, 5min

C. *E. coli* transformation: transform 5ul into E.coli using standard chemical transformation methods and plate onto LB+Carbenicillin for selection

Cloning-free stitching of gRNA expression cassettes (making the "C" fragment):

This protocol describes the process for stitching PCR amplification of a custom gRNA expression cassette that can be directly transformed into *C. albicans* <u>without the need for cloning</u>. The protocol is identical for each of the three CRISPR systems, with the exception of unique template plasmids for amplification of fragment "B", and a unique "reverse" oligo for amplification of fragment "C" (noted below).

E. PCR amplify the universal "A" fragment from pADH110 using AHO1096 and AHO1098

- PCR Mix (makes enough "A" fragment for >75 "C" fragment stitching PCRs):
1. 75.5ul H_2O
2. 20ul 5x Phusion HF buffer
3. 2ul dNTP mix (10 mM each dNTP)
4. 1ul pADH110 (1ng/ul)
5. 0.5ul AHO1096 (100uM)
6. 0.5ul AHO1098 (100uM)
7. 0.5ul Phusion polymerase

- "A" fragment PCR cycling conditions:
7. 98°C, 30sec
8. 98°C, 20sec
9. 58°C, 20sec
10. 72°C, 30sec
11. Return to step 2 for a total of 30 cycles
12. End

F. PCR amplify a unique “B” fragment from gRNA plasmid 1of2

i. PCR Mix (makes enough “B” fragment for >10 “C” fragment stitching PCRs):
1. 13.3ul H_2O
2. 4ul 5X Phusion HF buffer
3. 0.4ul dNTP mix (10 mM each dNTP)
4. 1ul AHO1097 (10uM)
5. 1ul custom gRNA oligo (10uM)
6. 0.2ul gRNA 2of2 plasmid (1ng/ul)
    - pADH119 for *C. alb* LEUpOUT
    - pADH139 for *C. mal* LEUpOUT
    - pADH147 for HIS-FLP
7. 0.1ul Phusion polymerase

- “B” fragment touchdown PCR cycling conditions:
1. 98°C, 30sec
2. 98°C, 20sec
3. 65°C, 20sec
4. 72°C, 30sec
5. Return to step 2 for a total of 10 cycles, reducing annealing temperature by 1°C each cycle
6. 98°C, 20sec
7. 55°C, 20sec
8. 72°C, 30sec
9. Return to step 6 for a total of 25 cycles
10. End

G. Stitching PCR to amplify unique “C” fragment gRNA expression cassette

- PCR Mix (makes enough “C” fragment for two transformations):
1. 74.5ul H_2O
2. 20ul 5x Phusion HF buffer
3. 2ul dNTP mix (10 mM each dNTP)
4. 1ul universal “A” fragment PCR
5. 1ul unique “B” fragment PCR
6. 0.5ul Phusion polymerase

- Stitching PCR cycling conditions (step 1):
1. 98°C, 30sec
2. 98°C, 20sec
3. 58°C, 20sec
4. 72°C, 1min
5. Return to step 2 for a total of 5 cycles
6. End

- After step 1, add amplification oligos
    - Add 0.5ul of 100uM AHO1237
    - Add 0.5ul of 100uM “reverse” oligo

- AHO1238 for *C. alb* LEUpOUT
- AHO1453 for *C. mal* LEUpOUT
- AHO1236 for HIS-FLP

- Stitching PCR cycling conditions (step 2):
  1. 98°C, 30sec
  2. 98°C, 20sec
  3. 66 °C, 20sec
  4. 72°C, 1min
  5. Return to step 2 for a total of 30 cycles
  6. End

Donor DNA design:

Donor DNA can be prepared by annealing synthetic DNA oligos or by PCR amplification of larger fragments. Annealed oligos are effective for introducing full-gene deletions or small-scale edits at the target locus. PCR amplified donor DNA can be used to incorporate larger DNA fragments at the target locus, including add-back of a gene at its native locus or introduction of non-native DNA constructs, such as promoter swaps, translational fusions, or overexpression constructs. We recommend using at least 45bp each of upstream and downstream flanking homology for synthetic dDNA fragments; shorter flanking sequences have been used successfully, but this tends to reduce the efficiency of targeted editing and thus necessitates increased colony PCR screening to identify colonies that carry the intended modification(s). For gene addback approaches, we recommend using 500bp of upstream and downstream homology to the target locus. When inserting non-native DNA at the target locus, such as creating promoter swaps or translational fusions, we recommend the use of "touchdown" PCR amplification with oligonucleotides that introduce ~50bp of upstream and downstream flanking homology to the target locus.

Donor DNA preparation:

- Annealed oligo donor DNA (makes enough for 5-10 transformations):

  Mix the following:
  1. 70ul H_2O
  2. 10ul "top" strand oligo (100uM)
  3. 10ul "bottom" strand oligo (100uM)
  4. 10ul 10x FastDigest Buffer (clear, not green)

  Anneal using "Quick Anneal" protocol (~15min total):
  1. 99°C 30sec
  2. Ramp down to 65 °C at a rate of 0.1°C per second
  3. End

- PCR-amplified donor DNA (makes enough dDNA for 1-2 transformations):

  Mix the following:
  1. 75.5ul H_2O

2. 20ul 5x Phusion HF buffer
3. 2ul dNTP mix (10 mM each dNTP)
4. 0.5ul “forward” primer (100uM)
5. 0.5ul “reverse” primer (100uM)
6. 1ul template DNA (1ng/ul for plasmid DNA, 10ng/ul for genomic DNA)
7. 0.5ul Phusion polymerase

PCR cycling conditions:

1. 98°C, 30sec
2. 98°C, 20sec
3. N°C, 20sec
   Note: Adjust the annealing temperature as needed to match the optimal Tm if using short oligos. If using long oligos to introduce flanking homology, we recommend the “touchdown” PCR method, as outlined in the fragment “B” amplification conditions above.
4. 72°C, 30sec/kb product length
5. Return to step 2 for a total of 30 cycles
6. End

C. albicans CRISPR transformation:

Day 1:

1. Inoculate 4ml YPD overnight(s) of strain(s) to be transformed and incubate at 30C with shaking

Day 2:

1. Dilute overnight culture(s) 1:50 in fresh YPD and incubate ~4-6hrs at 30°C until OD_{600} is between 0.5-0.8
   a. NOTE: 5ml of diluted culture is sufficient for 1 transformation, so scale accordingly
   b. NOTE: transformation efficiency decreases dramatically if the OD_{600} at harvest is ≥1
2. Pellet cells and wash 2x in sterile deionized water
   a. Transfer cells to a centrifuge tube and spin 5min at 4k rpm in table-top centrifuge
   b. Decant supernatant into liquid bio waste and resuspend cell pellet(s) in 1ml sterile water
   c. Transfer to a 1.75ml microfuge tube and pellet again by spinning 1min at 4k rpm in bench-top centrifuge (or ~30s in minifuge)
   d. Decant supernatant and resuspend cell pellet(s) in 1ml sterile water
   e. Pellet 1min at 4k rpm in bench-top centrifuge (or ~30s in minifuge) and decant supernatant
   f. Resuspend cell pellet in $1/100^{th}$ of original volume
      i. For 50ml of culture prepared in Day2 step 1, resuspend in 500ul sterile water
3. Mix the following components in a 1.7ml Eppendorf tube:
   a. 10ul 10mg/ml denatured salmon sperm DNA
      i. NOTE: make sure ssDNA is boiled and snap-cooled on wet ice prior to use. Can be stored at -20 and used directly after thawing without re-boiling.

b. 2ug of each MssI-digested CRISPR plasmid
    i. If using stitched gRNA fragment, add 50ul of fragment "C" PCR per transformation
c. 10ul of 10uM annealed donor DNA or 50ul of unpurified PCR amplified donor DNA
    i. Note: PCR amplified dDNA should yield a bright band if 10ul is checked on a gel
d. 50ul of washed cell suspension from Day2 step 2f
e. 1ml of freshly-prepared PLATE mix (make enough mix for N+1 transformations)
    i. 875uL 50% PEG 3350 (NOTE: make sure PEG solution is ≤ 2 months old)
    ii. 100uL 10X TE (100mM Tris pH 7.4, 10mM EDTA pH8)
    iii. 25uL 1M LiOAc, pH 7 (NOTE: make sure LiOAc solution is ≤ 2 months old and is pH'd with acetic acid)
f. <u>Mix by inversion</u> and gentle flicking to ensure <u>complete mixing</u>

4. Incubate overnight at 30 ^{o}C without shaking
    a. Remember to turn on the water bath at 44 ^{o}C

Day 3:

1. Heat-shock 15min in 44 ^{o}C water bath
2. Wash cells 1x with YPD
    a. Pellet 2min at 5k rpm in bench-top centrifuge and remove supernatant by aspiration
    b. Resuspend by pipette mixing in 1ml YPD (<u>use filter tips!</u>)
    c. Pellet 2min at 5k rpm in bench-top centrifuge and remove supernatant by aspiration
    d. Resuspend in 1mL YPD and proceed to step 3.
3. Allow cells to recover 5hrs in YPD before plating
    a. Transfer 1ml cell suspension from Day3 step 2d into 14ml snap-cap tubes with an additional 1ml YPD (2ml total)
    b. Transfer to 30 ^{o}C shaking incubator and incubate for <u>5hrs</u>
        i. We <u>do not</u> recommend longer recoveries, as this will decrease efficiency
    c. Pellet 2min at 5k rpm in table-top centrifuge then decant supernatant into liquid biological waste
    d. Resuspend pellet in residual YPD left in the tube and plate to YPD+NAT
4. Transfer plates to 30 ^{o}C incubator for 2-3days (or bench top for 3-4days)

Colony PCR verification of target locus modification:

Donor DNA integration (and the resulting modification of the target locus) can be confirmed by performing colony PCR. In the case of a gene deletion, ORF-specific primers and flank-specific primers should be designed (see figure 4). Each primer pair should amplify a product in the range of ~400-700bp, with the ORF-specific primers yielding a product only if the original ORF is present, while the flank-specific primers should yield a product of the expected size only if the ORF is deleted. If the target sequence has been modified, or exogenous DNA was introduced into the target locus, then integration-specific primers should also be designed as shown in figure 4. NOTE: If looking for deletion of a target locus, we recommend first screening for the presence of the modified locus, then streaking cPCR-positive isolates onto YPD+NAT (to obtain single-colony isolates) prior to using ORF-specific cPCR to confirm loss of the target locus. This re-streaking step is important to avoid any false-positives in the ORF-specific cPCR that can arise due to carryover of non-viable wild-type cells on the original transformation plate. It is also important to include a wild-type control strain in your ORF-specific cPCR to verify that the ORF-specific primers are functional.

Figure 4:
Colony PCR verification of modified target locus

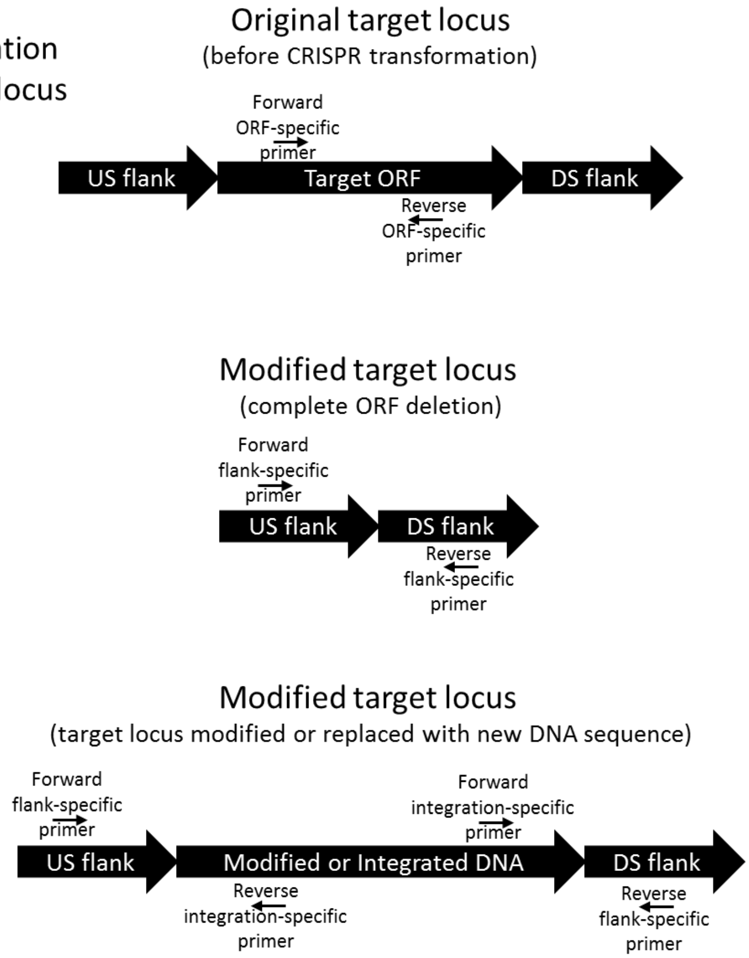

Preparation of colony lysate:

1. Prepare one PCR tube (or well in a PCR plate) containing 100ul of 20mM NaOH for each colony to be tested
2. For each colony to be tested, pick a small dab of the colony using a sterile tootpick and patch onto a YPD+NAT plate before swirling the toothpick into the NaOH
3. Cap and heat for 10min at 99°C

Preparation of colony PCR master mix (per reaction…scale up as needed):

1. 2.2ul 10x DreamTag Green Buffer
2. 4.4ul 5M Betaine
3. 0.44ul 50mM $MgCl_2$
4. 0.44ul dNTP mix (10mM each)
5. 0.2ul each oligo (100uM)
6. 0.18ul DreamTaq
7. 11.94ul H_2O
8. 2ul Lysate

Colony PCR cycling conditions:

1. 94°C, 30sec
2. 94°C, 20sec
3. 55°C, 20sec
4. 72°C, ~2min/kb product size
5. Return to step 2 for 30 total cycles
6. End

LEUpOUT removal of CRISPR components and NAT from the LEU ORF (Use with the LEUpOUT systems):

After confirming the intended modifications at the target locus, the CRISPR components and NAT marker can be removed from strains transformed with the LEUpOUT system via selection on –LEU media. Always confirm LEU^{+}/NAT^{S} phenotype after LEUpOUT, as some LEU+ colonies may retain NAT^{R}, and are presumed to be aneuploids.

1. After single-colony isolation of colony PCR-verified mutants on YPD+NAT, re-streak 2-4 independent colonies directly onto SD –LEU and incubate 2-3days at 30°C to isolate LEU+ single colonies.
2. Confirm removal of the NAT marker by patching LEU+ colonies onto YPD +/- NAT and grow overnight at 30°C.
    a. Note: we recommend YPD + 400ug/ml nourseothricin for +NAT patch plates
3. LEU+ NAT^{S} isolates are now ready for strain banking and subsequent analysis.

FLiP-out removal of CRISPR components and NAT marker (use with the HIS-FLP system):

The CRISPR components and NAT marker can be removed from strains transformed with the HIS-FLP system via induction of FLP recombinase in YP-Maltose media and subsequent screening for NAT^{S} isolates.

1. After single-colony isolation of colony PCR-verified mutants on YPD+NAT, inoculate 1-2 independent colonies into 3ml YP maltose liquid media and culture ~16hrs with shaking at 30°C.
2. Dilute cells to ~1,000cfu/ml, plate 100ul onto YPD+NAT^{25}, and incubate 2-3days at 30°C (or until it is possible to differentiate between large and small colonies).
3. Patch small to medium sized colonies onto YPD +/- NAT and grow overnight at 30°C.
    a. Note: we recommend YPD + 400ug/ml nourseothricin for +NAT patch plates
4. NAT^{S} isolates are now ready for strain banking and subsequent analysis.
