# Supplementary material for: An Efficient, Rapid, and Recyclable System for CRISPR-Mediated Genome Editing in Candida albicans
Source: mSphere. 2017 Apr 26;2(2):e00149-17. doi: 10.1128/mSphereDirect.00149-17 (PMC5422035; doi:10.1128/mSphereDirect.00149-17)
Supplement: FIG S2 [file sph002172275sf8.pdf]

Figure S2A

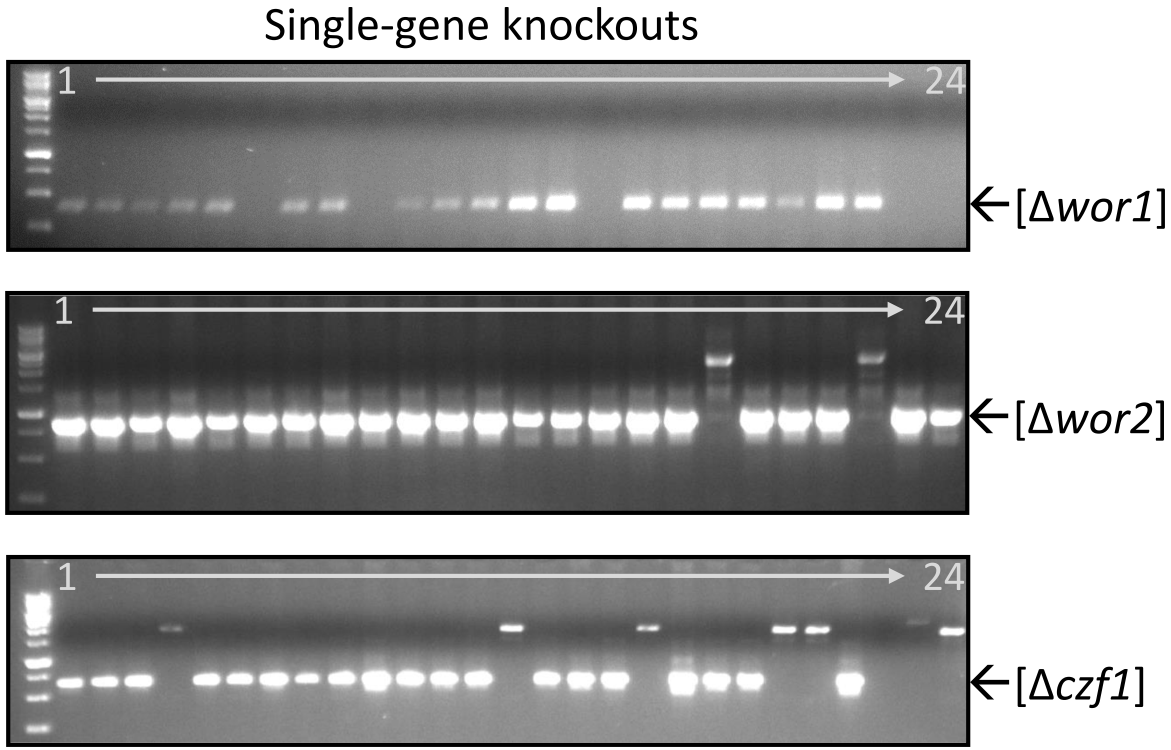

Figure S2B

WOR1 + *WOR2* double-double knockouts

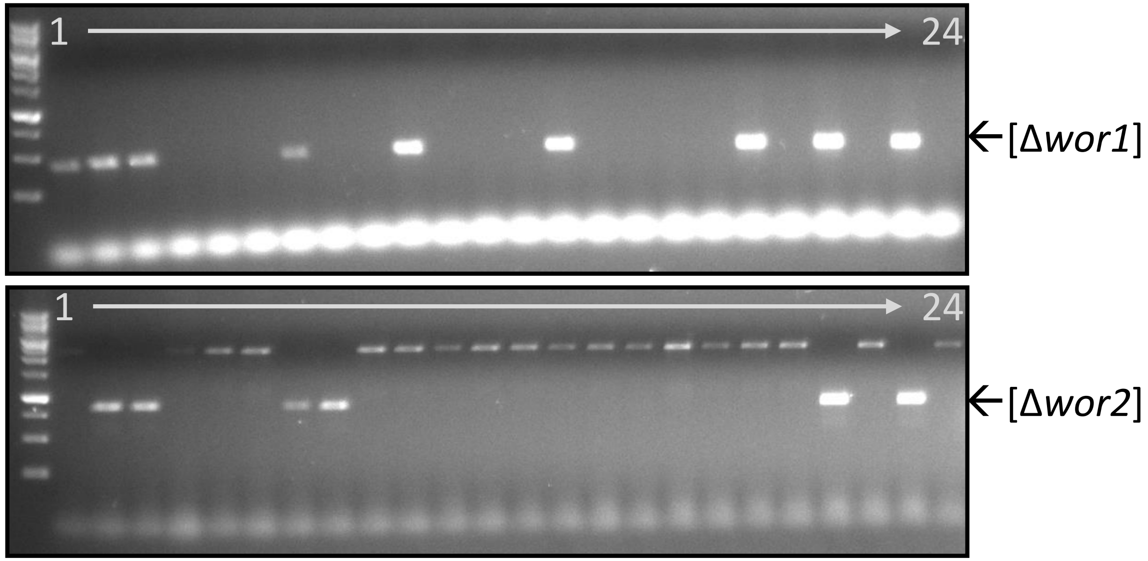

WOR2 + *CZF1* double-double knockouts

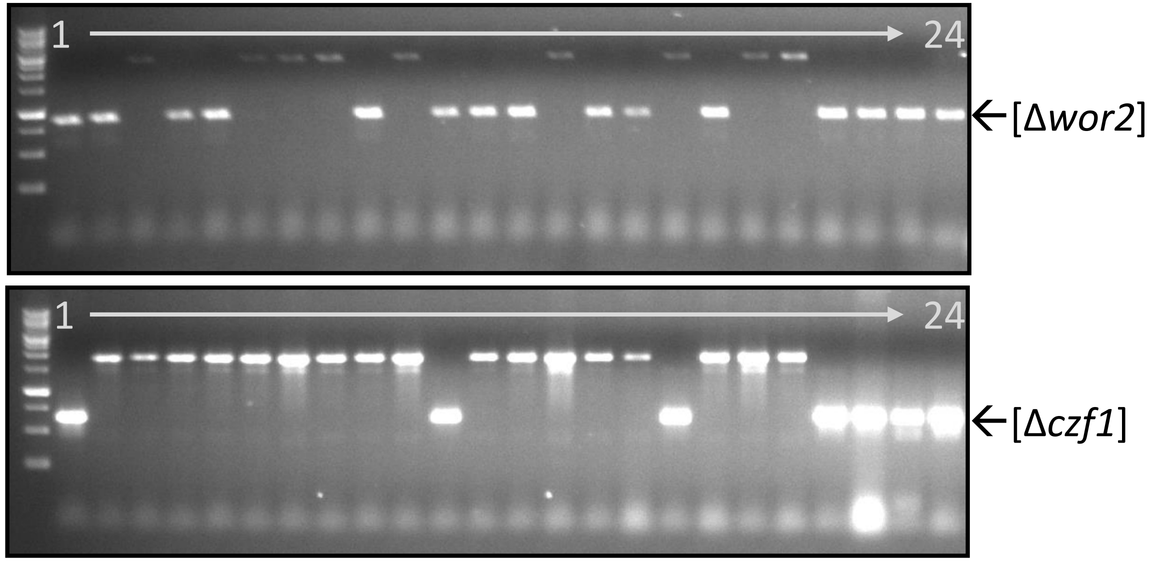

Figure S2C

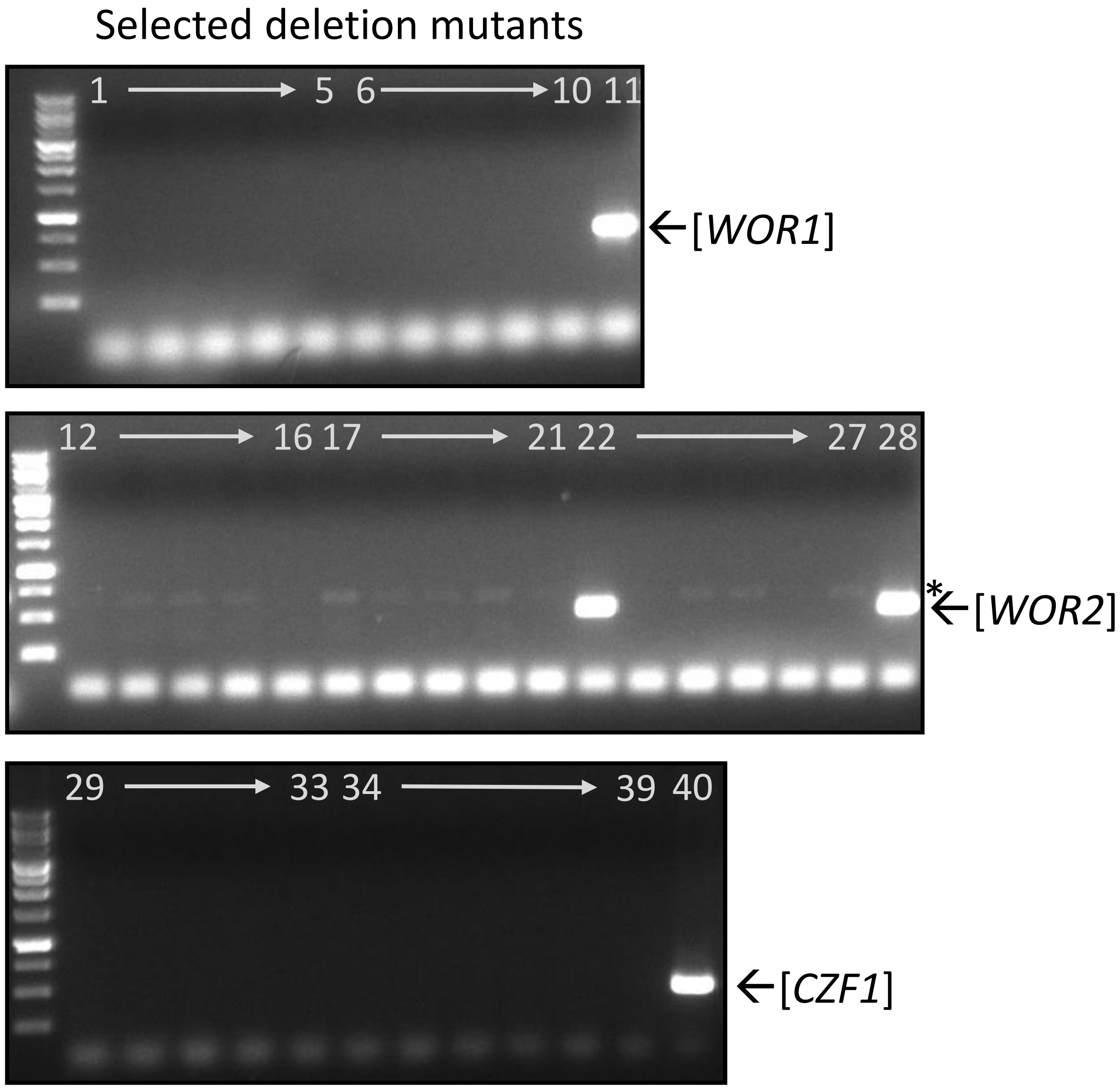

Selected deletion mutants
1 → 5 6 → 10 11
←[WOR1]
12 → 16 17 → 21 22 → 27 28
*
←[WOR2]
29 → 33 34 → 39 40
←[CZF1]
